# The mechanism of a chitosan-collagen composite film used as biomaterial support for MC3T3-E1 cell differentiation

**DOI:** 10.1038/srep39322

**Published:** 2016-12-21

**Authors:** Xiaoyan Wang, Gan Wang, Long Liu, Dongyi Zhang

**Affiliations:** 1 Department of Chemistry and Biology, College of Science, National University of Defense Technology, Changsha, Hunan, 410073, PR China

## Abstract

Natural composite biomaterials are good structural supports for bone cells to regenerate lost bone. Here, we report that a chitosan-collagen composite film accelerated osteoblast proliferation, differentiation and matrix mineralization in MC3T3-E1 cells. Intriguingly, we observed that the film enhanced the phosphorylation of Erk1/2. We showed that the chitosan-collagen composite film increased the transcriptional activity of Runx2, which is an important factor regulating osteoblast differentiation downstream of phosphorylated Erk1/2. Consistent with this observation, we found that the chitosan-collagen composite film increased the expression of osteoblastic marker genes, including *Type I Collagen* and *Runx2* in MC3T3-E1 cells. We conclude that this film promoted osteoblast differentiation and matrix mineralization through an Erk1/2-activated Runx2 pathway. Our findings provide new evidence that chitosan-collagen composites are promising biomaterials for bone tissue engineering in bone defect-related diseases.

Previous studies showed that the extracellular signal-regulated kinase (Erk) pathway is one of the vital pathways during osteoblast differentiation and is also vital for many cellular responses. Multiple signals, such as growth/hormone factor stimulation and matrix-integrin binding, activate the Erk pathway in bone. Activated Erk promotes calvarial osteoblast differentiation through the downstream transcription factor Runx2 at Ser^301^ and Ser^319^ residues, and the mRNA levels of specific osteoblast differentiation-related marker genes are increased^1^,^2^,^3^,^4^,^5^,^6^.

The treatment of bone defects, due to trauma, infections, tumours, or genetic malformations, is a major challenge for clinicians. Bone tissue engineering has been widely studied with the goal of treating bone defects. Composite biomaterials, especially natural composite materials, provide good structural support for bone cells to regenerate the lost bone and stimulate osteoblast proliferation, differentiation and mineralization^7^. They also mimic the properties of matrix proteins to modulate the process of bone formation^8^. Chitosan and collagen are two natural materials that are commonly used in bone tissue engineering^9^,^10^,^11^.

Chitosan [β-(1,4)-2-amino-2-deoxy-D-glucan], which is the N-deacetylated derivative of chitin, is abundant in nature. It has been widely used in controlled release drugs, artificial skin, bone disease treatment and surgical sutures in the pharmaceutical and medical sciences due to its good biocompatibility, biodegradability and low toxicity^12^. It can be made in various forms, such as a film, fibre, a porous scaffold or a tube^13^. Chitosan composites with collagen, gelatine, hyaluronic acid (HA), calcium phosphate, polymethylmethacrylate (PMMA), alginate and poly L-lactic acid (PLA) have been used during the process of bone tissue engineering^14^,^15^,^16^.

Collagen, which is the most abundant protein in mammals, makes up 25% to 35% of the whole-body protein content and represents the major constituent of animal cartilage and connective tissue. It is widely used as the scaffold during bone regeneration due to its outstanding cell adhesion, proliferation and differentiation properties. Collagen has been reported to promote MC3T3-E1 cell proliferation^17^,^18^ and enhance stem cell differentiation into osteoblasts^19^. However, it is difficult to use alone as a biomaterial because of its low mechanic properties and easy degradation during bone tissue engineering.

Previous studies showed that a chitosan-collagen composite made using different methods can be used as a tissue engineering material. Chitosan has a lower degradation rate and greater mechanical properties, whereas collagen has better cellular affinity as a biomaterial. Blended films with different chitosan and collagen mass ratios significantly affected the cell morphology, adhesive force and Young’s modulus^20^. Stem cells could be cultured on chitosan-collagen formed sponges^21^. The process of bone marrow mesenchymal stem cell development, proliferation and osteogenic differentiation was enhanced after culturing on chitosan hydrogel and collagen^22^. Many studies have shown that chitosan and collagen can be used in bone tissue engineering to influence osteoblast proliferation, differentiation and mineralization. While previous studies focused on the overall cell performance, little is known about the molecular mechanism of the cells when cultured on these biomaterials. Therefore, it is important to characterize the molecular mechanism of the cell behaviour when the cells are cultured on chitosan and collagen composites to achieve better use in bone tissue engineering.

In this study, different mass ratios of chitosan and collagen were used to prepare composite films, and we investigated the mechanism of chitosan-collagen composite film mediation of MC3T3-E1 cell differentiation. Our findings provide the molecular mechanism of MC3T3-E1 cells cultured on the chitosan-collagen composite film, a potential bone tissue engineering biomaterial.

## Results

### Characteristics of chitosan-collagen composite films

**Figure 1:**
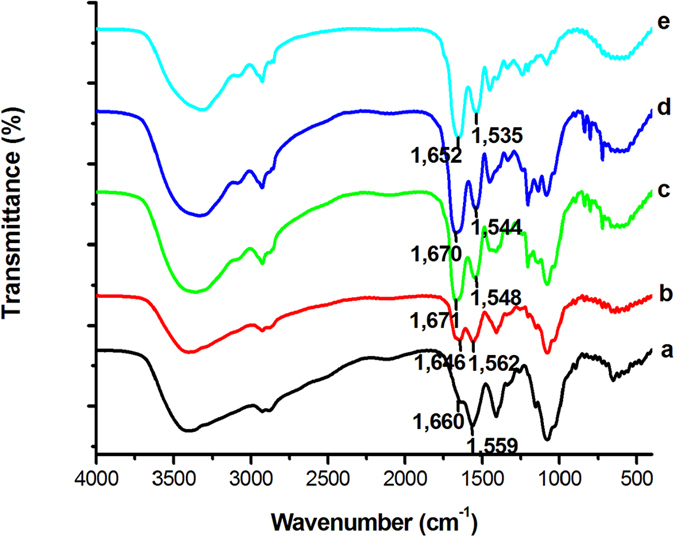
Characterization of chitosan-collagen composite films. FTIR spectra of the chitosan-collagen composite films: (**a**) 0 collagen film; (**b**) 0.25 collagen film; (**c**) 0.5 collagen film; (**d**) 0.75 collagen film; (**e**) collagen film.

To study the role of chitosan-collagen composite films on osteoblasts, we used a Fourier transform infrared spectroscopy (FTIR) spectrometer to illustrate their characteristics. The FTIR spectrum of the chitosan film had a characteristic amino group (-NH_2_) peak at 1559 cm^−1^ (Fig. 1a). The amide peak intensity for chitosan corresponded to the partial N-deacetylation in chitin. The characteristic peak of collagen was observed at 1652 cm^−1^, corresponding to the carboxyl group (-COOH) in the molecular chain (Fig. 1e). The FTIR spectra of chitosan-collagen composite films are shown in Fig. 1. Incorporation of collagen led to small changes in the spectrum of chitosan. The peaks from both the carbonyl and amino groups were both shifted, and the intensity of the peaks also changed. This result implies that a hydrogen bond was formed between the chitosan and collagen molecules.

### Chitosan-collagen composite films promoted osteoblast proliferation, differentiation and mineralization *in vitro*

**Figure 2:**
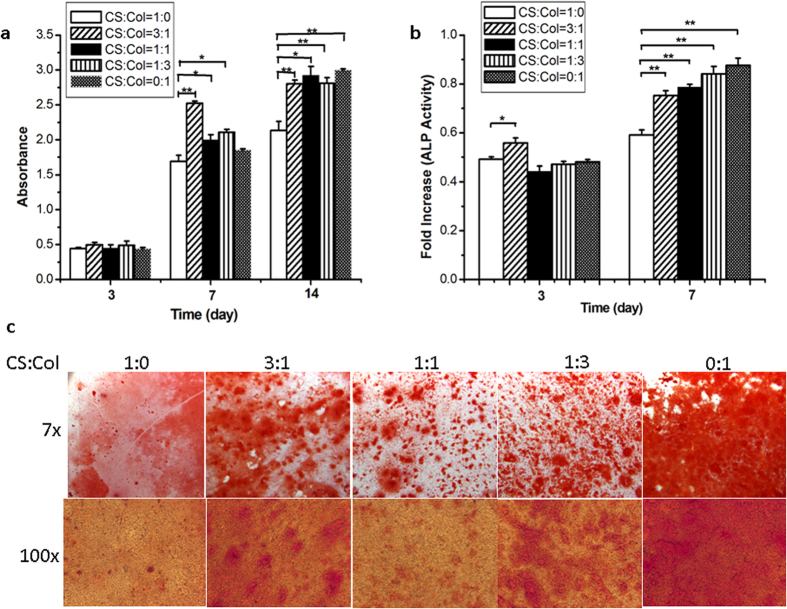
Chitosan-collagen composite films influenced MC3T3-E1 cell proliferation, differentiation and matrix mineralization. (**a**) CCK-8 assay of the MC3T3-E1 cells plated on chitosan-collagen composite films after culturing for 3, 7, and 14 days. (**b**) ALP activity of the MC3T3-E1 cells plated on chitosan-collagen composite films after culturing for 3 and 7 days. (**c**) Calcium mineralization of MC3T3-E1 cells plated on chitosan-collagen composite films after culturing for 14 days. Microscopic observations of mineralization were indicated using Alizarin red staining (top, 7X; bottom, 100X). Mineral nodules were stained with an Alizarin red solution. Data are expressed as the mean ± SE from all experiments, as indicated. *p < 0.05; **p < 0.01.

To investigate the effect of chitosan-collagen films on osteoblast proliferation, differentiation and matrix mineralization, we cultured MC3T3-E1 cells on chitosan-collagen composite films and induced the cells with a mineralization medium for osteoblast differentiation. We assessed osteoblast proliferation by measuring cell activity. The cell activity was significantly greater on the 0.25, 0.5 and 0.75 collagen films than on chitosan film at different time points (Fig. 2a). These data indicate that the collagen in the composite films facilitated MC3T3-E1 cell proliferation.

Then, we determined the differentiation ability of osteoblasts by measuring the activity of alkaline phosphatase (ALP), which is an early marker for osteoblast differentiation. ALP activity was significantly greater on the 0.25 collagen films than that on chitosan film at different time points, and it was also significantly greater on the 0.5, 0.75 and 1 collagen films 7 days after culturing. The ALP activity was greater on day 7 than on day 3 in all chitosan-collagen composite films (Fig. 2b). These data suggest that the films containing collagen enhance MC3T3-E1 cell differentiation.

Next, we observed mineralized nodules by staining the cells with an Alizarin red solution. Mineralized nodules were observed on day 14 after induction of differentiation using the mineralization medium. More mineralized nodules were observed on the 0.25, 0.5, 0.75 and 1 collagen films (Fig. 2c). These results indicate that films containing collagen enhance mineralization nodule formation *in vitro*. Taken together, our data suggest that films containing collagen enhance osteoblast differentiation.

### Chitosan-collagen composite films enhanced Erk phosphorylation and Runx2 activity

**Figure 3:**
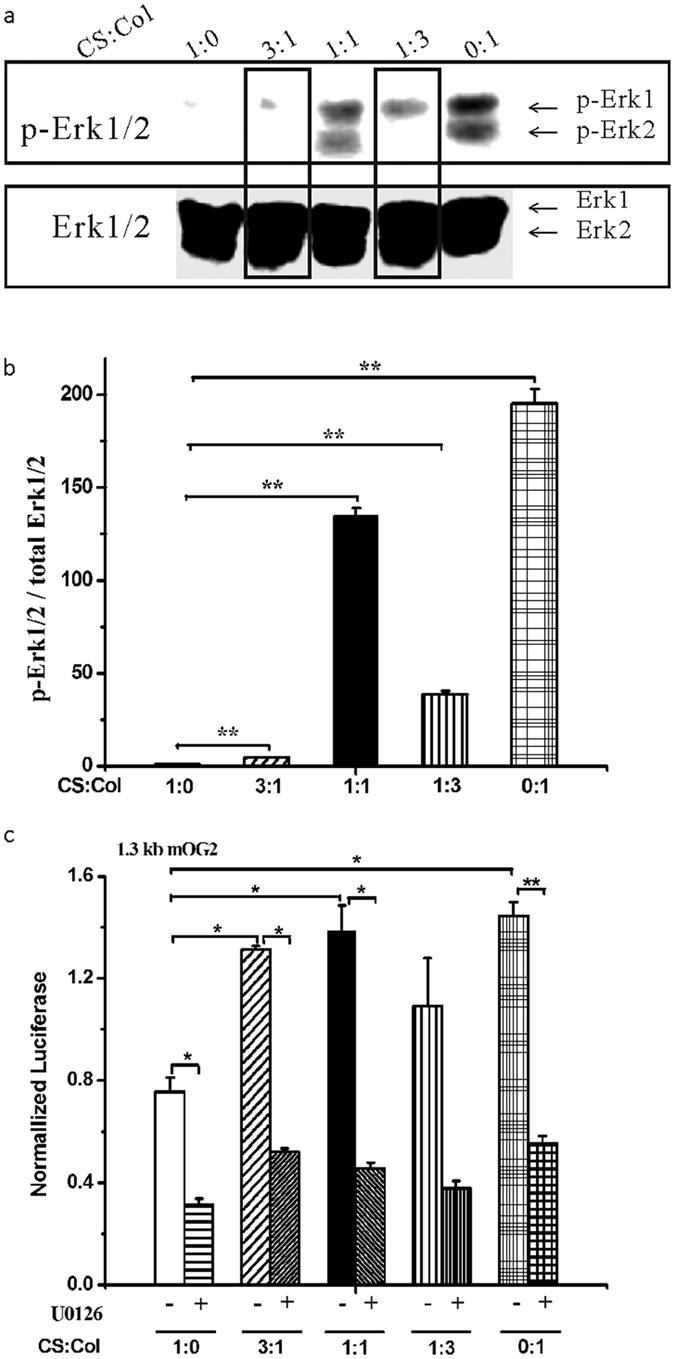
Chitosan-collagen composite films affected Erk phosphorylation and Runx2 transcriptional activity in MC3T3-E1 cells. (**a**) Erk1/2 phosphorylation in MC3T3-E1 cells. MC3T3-E1 cells plated on chitosan-collagen composite films and cultured for 6 days. A western blot was used to show phosphorylated Erk1/2, and the total Erk1/2 protein was used as a loading control. (**b**) Relative ratio of p-Erk1/2/Erk1/2 from the results in (**a**). (**c**) Runx2 activity in MC3T3-E1 cells. MC3T3-E1 cells plated on chitosan-collagen composite films were transfected with 1.3 kb mOG2-Luc plasmids in culture with αMEM. MC3T3-E1 cells were treated with U0126 for 24 hours. The Renilla luciferase plasmid pRL-SV40 was used as an internal control. Data are presented as the mean ± SE from three repeated experiments. A statistical analysis was performed as indicated. *p < 0.05; **p < 0.01.

To determine whether chitosan-collagen composite films affect the phosphorylation of Erk based on the fact that osteoblast differentiation requires Erk signaling, we detected the phosphorylation level of Erk1/2 in MC3T3-E1 cells on the chitosan-collagen composite films. The result indicated that after 6 days, Erk1/2 phosphorylation in the MC3T3-E1 cells was increased when the film contained collagen (Fig. 3a and b).

Runx2 is a transcription factor that is directly activated by the Erk signaling pathway and binds to DNA at the 1.3 kb mOG2 fragment^23^. We examined the activity of Runx2 by measuring a 1.3 kb mOG2-Luc reporter in which a minimal 1.3 kb mOG2 promoter was linked to the luciferase reporter gene. The Runx2 transcriptional activity was increased significantly on the collagen composite film as determined by assaying the 1.3 kb mOG2-Luc (Fig. 3c) reporter in a general medium, and the Runx2 transcriptional activity was increased significantly on the 0.25, 0.5, and 1 collagen films as determined by assaying the 1.3 kb mOG2-Luc reporter. The Runx2 activity was decreased on the composite films treated with Erk1/2 inhibitor U0126 (Fig. 3c). These results indicate that the films containing collagen had the ability to facilitate Runx2 transcriptional activity.

### Chitosan-collagen composite films increased osteoblast-specific gene expression

**Figure 4:**
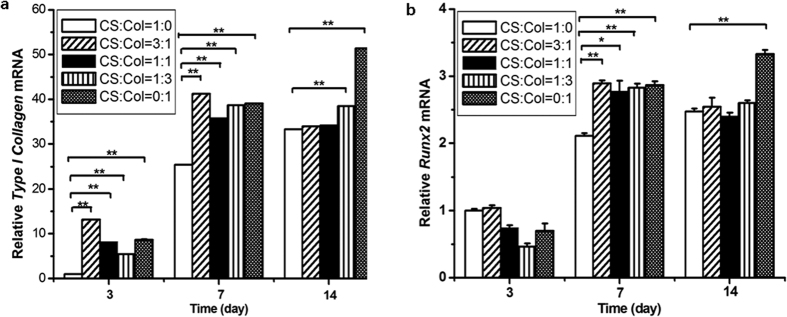
Chitosan-collagen composite films affected mRNA levels of osteoblast-specific genes in MC3T3-E1 cells. (**a**,**b**) chitosan-collagen composite films increased mRNA levels after incubation for 3, 7 and 14 days. *Type I Collagen* (**a**) and *Runx2* (**b**). Data from panels a to b are expressed as the mean ± SE from all experiments, as indicated. *p < 0.05; **p < 0.01.

Finally, we detected mRNA levels of osteoblast-specific genes in the MC3T3-E1 cell cultures on the chitosan-collagen composite films. *Type I Collagen* (Fig. 4a) and *Runx2* (Fig. 4b) were significantly increased indicating that the chitosan-collagen composite films promote osteoblast-specific gene expression. Taken together, all the data suggest that chitosan-collagen composite films increase the expression of osteoblast-specific genes at the transcriptional level through the Erk1/2 pathway in MC3T3-E1 cells.

## Discussion

Previous studies showed that there is a molecular interaction between chitosan and collagen in chitosan-collagen composites^24^, and this composite film affected osteoblast morphology, the adhesive force and the Young’s modulus in MC3T3-E1 cells^20^. Indeed, we observed that a hydrogen bond may be formed between the chitosan and the collagen molecules in the composite (Fig. 1). Intriguingly, we found that MC3T3-E1 cell cultured on composites containing collagen had an elevated phosphorylated level of Erk. Importantly, because phosphorylated Erk regulates Runx2 transcriptional activity by translocating to the nucleus^23^, we propose that chitosan-collagen composites can modulate osteoblast differentiation. Through the use of cell differentiation, matrix mineralization and a luciferase assay, we demonstrated that chitosan-collagen composite films enhanced osteoblast differentiation and matrix mineralization. Furthermore, the expression levels of osteoblastic marker genes, including *Type I Collagen* and *Runx2*, were increased significantly in the MC3T3-E1 cells that were cultured on the chitosan-collagen composite films. All these data suggest that chitosan-collagen composite films enhance osteoblast differentiation.

Osteoblasts undergo cell proliferation, differentiation and mineralization stages during their lives. In our study, the response of cells cultured on the composite films was also detected. The cell adhesive force was greater on collagen than on chitosan, and the adhesive force of chitosan-collagen composite film ranged between chitosan and collagen allowed the cells to adhere and spread quickly^20^. Our data showed that the chitosan-collagen composite films promoted MC3T3-E1 proliferation at day 7 and day 14 (Fig. 2a), which is consistent with previously reported results^20^. To our knowledge, this result is the first evidence that chitosan-collagen composite films promote Erk phosphorylation during osteoblast differentiation (Fig. 3a and b). Because phosphorylated Erk, which is essential for osteoblast differentiation, regulates the transcriptional activity of Runx2 through translocation to the nucleus, we propose that chitosan-collagen could regulate osteoblast differentiation and matrix mineralization. By determining ALP activity and observing mineralized nodules, we demonstrated that chitosan-collagen composite films promoted osteoblast differentiation and mineralization (Fig. 2b and c). Furthermore, the transcriptional activity of Runx2 was significantly increased in the cells cultured on the chitosan-collagen composite films (Fig. 3c), and Runx2 activity was decreased after treatment with U0126 (Fig. 3c). These results suggest that MC3T3-E1 cells cultured on chitosan-collagen composite film have enhanced osteoblast differentiation and mineralization through the Erk-activated Runx2 pathway. We also provided data demonstrating that the expression levels of *Type I Collagen* and *Runx2*, which are osteoblastic marker genes, were also increased significantly (Fig. 4a and b). The data above suggest that chitosan-collagen composite films enhance osteoblast differentiation.

Erk-Runx2 and Bmp-Smad are two important pathways during osteoblast differentiation. Bmps binds to Bmp type I receptor to phosphorylate Smad1/5/8 or Erk1/2 in the nucleus. However, Erk1/2 phosphorylation can be rapidly activated by Bmps, and this process is independent of Smad1/5/8^25^,^26^. Erk phosphorylation has been reported to promote osteoblast differentiation through the Runx2 transcriptional factor, and activated Runx2 promotes osteoblast marker gene expression^27^,^28^. In this study, we found that chitosan-collagen composite films activated Erk phosphorylation and then promoted the transcriptional activity of Runx2 and downstream gene expression. We concluded that chitosan-collagen composite films may enhance osteoblast differentiation through regulation of Erk phosphorylation. However, we could not observe Smad1/5/8 phosphorylation (data not shown), indicating that the Bmp-Smad pathway is not activated in this process. Furthermore, collagen I could also induce Erk activation directly^29^. Therefore, the collagen in the composite films may activate Erk directly.

There are two questions that remain unclear. First, what percentage of collagen in the composite film has the best effect on osteoblast differentiation and matrix mineralization? Second, what types of chitosan-collagen composite structure regulate osteoblast differentiation and matrix mineralization? In our study, we observed that the composite films had an effect on osteoblasts because they contained different mass ratios of collagen, which has been reported to be important for matrix formation^30^. However, from our data, the effect of the chitosan-collagen composite film was not strictly dependent on the percentage of collagen. However, the trend of the effect was dependent on the percentage of collagen. There should be a specific collagen percentage in the chitosan-collagen composite that results in the best effect on the MC3T3-E1 cells. Therefore, we will perform additional experiments to determine this precise percentage of collagen in the composite film in future. It is also still unclear what structure of the chitosan-collagen composite is the best form for bone tissue engineering. Because a three-dimensional structure, which is known as a “scaffold”, could modulate bone formation by stimulating osteoblast proliferation and differentiation due to the structural support^31^, we speculate that a chitosan-collagen composite scaffold would be better than a film as a bone tissue engineering material. Further studies are required to test the role of chitosan-collagen composite scaffolds in bone formation.

In conclusion, we have described the role of a chitosan-collagen composite film in modulating osteoblast differentiation and matrix mineralization. Interestingly, we observed that the function of the chitosan-collagen composite film in osteoblast differentiation depended on the Erk pathway. We concluded that the chitosan-collagen composite film promoted osteoblast differentiation and matrix mineralization through the Erk-activated Runx2 signaling pathway. We believe that a chitosan-collagen composite may be a promising biomaterial for bone tissue engineering for bone defect-related diseases.

## Methods

### Materials and substrate preparation

Chitosan (degree of deacetylation = 80–95%) was purchased from Sinopharm Chemical Reagent Co., Ltd (Shanghai, China). Collagen was purchased from Sigma Company.

Chitosan (10 mg/ml in 1% v/v, acetic acid) and collagen (1 mg/ml in 1% v/v, acetic acid) solutions were added to dishes to prepare the thin films at 50 °C, and the mass ratios of chitosan-collagen blend films were 1:0, 3:1, 1:1, 1:3 and 0:1. Films were treated with 0.25 M NaOH for 20 min and were then washed three times with distilled water. Subsequently, the films were rinsed with ethanol overnight and washed three times with phosphate buffered saline (PBS).

### FTIR spectra

FTIR spectra were obtained using a Nicolet Avatar 360 system. The spectra were collected over the range of 400–4000 cm^−1^.

### Plasmids, cell line and cell culture

The reporter plasmid for Runx2 activity, 1.3 kb mOG2-Luc, was kindly provided by Dr. Franceschi RT (School of Dentistry, University of Michigan, USA). MC3T3-E1 cells were maintained in alpha minimum essential medium (αMEM) (Gibco, USA) supplemented with 10% foetal bovine serum (FBS), 100 units/ml of penicillin, and 100 μg/ml of streptomycin. The mineralization medium was prepared through the addition of 50 μg/mL ascorbic acid, 10 mM sodium β-glycerophosphate, and 10 nM dexamethasone into the αMEM.

### Cell proliferation

MC3T3-E1 cells were cultured on chitosan-collagen composite films in 96-well plates at a density of 2.5 × 10^4^ cells/well for 3, 7 and 14 days. Cell proliferation assays were measured by incubation using a Cell Counting Kit-8 (CCK-8) for 4 hours at 37 °C and reading at an absorbance of 450 nm.

### ALP activity

MC3T3-E1 cells were cultured on chitosan-collagen composite films in 96-well plates at a density of 2.5 × 10^4^ cells/well for 3 and 7 days. Cell lysates from the same treatment were divided into two parts to measure the protein concentration and the ALP activity. A BCA protein assay kit (Pierce, USA) was used to determine protein concentration at an absorbance of 550 nm. The ALP assays were performed after incubation in 0.1 M NaHCO_3_-Na_2_CO_3_ buffer (pH 10.0) containing 0.1% Triton X-100, 2 mM MgSO_4_, and 6 mM *p*-nitrophenol inorganic phosphate (PNPP) for 30 minutes at 37 °C at an absorbance of 405 nm. The value of the absorbance at 405 nm divided by the value of the absorbance at 550 nm (A_405_/A_550_) was used to calculate the relative ALP activity.

### Mineralization analysis

MC3T3-E1 cells were cultured on chitosan-collagen composite films in a 6-well dish at a density of 2.5 × 10^4^ for osteoblast differentiation. After the cells were incubated for 3 days, the medium was changed to mineralization medium. The medium was changed every 3 days. After incubation for 14 days, mineralization was examined using Alizarin red staining. The cells were fixed in 70% ethanol for 10 minutes, and the calcium deposits were stained for 15 minutes with the Alizarin red solution (40 mM, pH 4.2) at room temperature. Several washes in water removed the nonspecific staining.

### Western blotting

MC3T3-E1 cells were harvested and washed 3 times with ice-cold PBS and lysed with TEN-T buffer (150 mM NaCl, 10 mM Tris- HCl, pH 7.4, 5 mM EDTA, pH 8.0, 1% Triton X-100, 1 mM PMSF, and 2 μg/mL of aprotinin). Cell debris was discarded by centrifuging at 12,000 × *g* for 15 minutes at 4 °C. Using 10% sodium dodecyl sulfate (SDS)-polyacrylamide gels, 30 μg of total protein was fractioned. Proteins were transferred to polyvinylidene fluoride (PVDF) membranes for blotting. Proteins were blocked with 5% skim milk for 2 hours at room temperature. The antibody against p-Erk1/2 was used at a 1:1000 dilution (Cell Signaling Technology, USA) and Erk1/2 at 1:1000 (Beyotime Biotech, China) for blotting overnight at 4 °C. Secondary antibodies were used at 1:10,000 dilutions. An enhanced chemiluminescent substrate for the detection of horseradish peroxidase (HRP) (Pierce, USA) was used to visualize the immunoreactivity. Molecular analyst software (Quantity One, Bio-Rad, USA), was used to detect the density of the areas of interest in the western blotting experiment. Erk1/2 was used as an internal control. The density of p-Erk1/2/Erk1/2 was computed and represented.

### Cell transfection and luciferase assay

MC3T3-E1 cells were transfected using lipofectamine 2000 (Invitrogen, USA) according to the manufacturer’s instruction. Cells were cultured on chitosan-collagen composite films in a 24-well dish at a density of 1 × 10^5^ for transfecting plasmids into the cells. The cells were washed twice with αMEM without FBS, penicillin or streptomycin and were incubated with 400 μl αMEM. Plasmids (0.5 μg 1.3 kb mOG2-Luc and 0.01 μg Renilla luciferase plasmid) were added to 50 μl of αMEM. A 2.5 μl aliquot of the lipofectamine 2000 reagent was diluted in 50 μl of αMEM. After incubating the dilutions at room temperature for 5 minutes, the diluted lipofectamine 2000 reagent was added to the plasmid mixture. After incubation for 20 minutes at room temperature, the mixtures were added to the cells. The medium was changed to normal medium after 6 hours. A Dual Luciferase Assay Kit (Beyotime, China) was used to measure the luciferase activities on a Fluoroskan Ascent FL (Thermo scientific, USA) 2 days after transfection. Cells were washed once with ice-cold PBS, and lysis buffer was used to lyse the cells for 15 minutes. Luciferin buffer was added to the cell lysates to detect the firefly luciferase, and then, coelenterazine buffer was added to the cell lysates to detect the renilla luciferase as the internal control. The value of firefly luciferase divided by the value of the renilla luciferase was used to determine the relative activity of the luciferase reporter gene.

### Quantitative real-time polymerase chain reaction (RT-qPCR)

MC3T3-E1 cells were lysed with Trizol reagent (Invitrogen, USA) and incubated at room temperature for 5 minutes. After shaking with chloroform, cell lysates were centrifuged at 12,000 × *g* for 15 minutes at 4 °C. The total RNA in the upper aqueous phase was placed into a new tube. Isopropanol was added to the aqueous phase at room temperature for 10 minutes. After centrifuging at 12,000× *g* for 10 minutes at 4 °C for RNA precipitation, the total RNA was washed with 75% ethanol. The RNA was air dried and resuspended in RNase-free water for cDNA synthesis. Oligo dTs, dNTPs and total RNA were added into one tube. After incubation at 65 °C for 5 minutes, the tube was kept on ice for at least 1 minute. After adding first-strand buffer, dithiothreitol (DTT), RNase inhibitor and SuperScript III (Invitrogen, USA), the tube was incubated at 50 °C for 1 hour following incubation at 70 °C for 15 minutes to obtain cDNA. cDNA was subsequently used for RT-qPCR using SYBR green (Applied Biosystems, USA). The following forward and reverse primers were used: *Gapdh* 5′-CATGGCCTTCCGTGTTCCTA-3′ and 5′-CCTGCTTCACCACCTTCTTGAT-3′; *Type I Collagen* 5′-CCTGGTAAAGATGGTGCC-3′ and 5′-CACCAGG TTCACCTTTCGCACC-3′; and *Runx2* 5′-GAATGCACTACCCAGCCAC-3′ and 5′-TGGCAGGTACGTGTGGTAG-3′. Amplifications were performed in a programme for 45 cycles as follows: first cycle (10 minutes at 95 °C, 1 minute at 56 °C, 30 seconds at 72 °C) and the next 44 cycles (30 seconds at 95 °C, 1 minute at 56 °C, 30 seconds at 72 °C).

### Statistical analysis

Data are expressed as the mean ± SE from at least three independent experiments. Significance was subject to an unpaired Student’s *t* test. A *p* value of less than 0.05 was used for a threshold of significance.

## Additional Information

**How to cite this article**: Wang, X. *et al*. The mechanism of a chitosan-collagen composite film used as biomaterial support for MC3T3-E1 cell differentiation. *Sci. Rep.* **6**, 39322; doi: 10.1038/srep39322 (2016).

**Publisher's note:** Springer Nature remains neutral with regard to jurisdictional claims in published maps and institutional affiliations.
